# Personal

**Published:** 1900-04

**Authors:** 


### PERSONAL.

DR. HORTENSE V. BRUCE, who since August, 1897, has been the resident physician at Westminster House, on Adams Street, Buffalo, has been appointed superintendent of the House of Refuge for Women at Hudson, N.Y. Dr. Bruce was graduated from the Medical Department of the University of Michigan in 1897. For six months she was interne in the prison for women at Sherburn, Mass. Her work among the poor and unfortunate, coupled with wide knowledge and excellent executive ability, admirably fit her for the difficult position she is called to fill.

---

DR. CHARLES G. STOCKTON, of Buffalo, was tendered a reception by the Medical Club of Philadelphia, at the Hotel Bellevue, in that city on the evening of March 8, 1900. The JOURNAL is pleased to acknowledge the receipt of an invitation, and regrets that it was prevented from attending to do honor to our distinguished Buffalonian.

---

DR. F. PARK LEWIS, of Buffalo, has been nominated by Governor Roosevelt to be a trustee of the State School for the Blind, at Batavia.

Dr. Lewis has already served in that capacity, hence will return to the board with a valuable experience.

---

Dr. Roswell Park, of Buffalo, who has been ill from influenza for some weeks is recovering, and it is anticipated that he will soon resume his accustomed collegiate and professional duties.

---

Dr. Julius H. Potter, of Buffalo, who lately underwent an operation for appendicitis at the Riverside Hospital, is said to be improving, with the prospects of early recovery.

